# SEX DIFFERENCES IN MULTI-DIMENSIONAL SLEEP HEALTH AND DEMENTIA RISK: A PROSPECTIVE STUDY IN THE UK BIOBANK

**DOI:** 10.1093/geroni/igae098.4359

**Published:** 2024-12-31

**Authors:** Tianyi Huang, May Beydoun, Lenore Launer

**Affiliations:** National Institute on Aging, Baltimore, Maryland, United States; National Institute on Aging, Baltimore, Maryland, United States; National Institute on Aging, Baltimore, Maryland, United States

## Abstract

Sex differences in sleep health risk factors may contribute to differences in dementia risk between women and men. There were 409,789 dementia-free UK Biobank participants (55% women) who self-reported 7 sleep-related traits at baseline in 2006-2010. Incident dementia was identified using algorithmically defined outcomes through primary care, hospital admissions, or death records until December 31, 2022. The prevalence of insomnia symptoms and non-restorative sleep was 7-8% higher in women, while snoring, sleepiness and napping were more common in men. Over a median follow-up period of 13.8 years, there were 7,583 incident dementia cases (3,406 Alzheimer’s, 1,651 vascular). After adjusting for potential confounders, the increased dementia risk associated with sleepiness, napping, and evening chronotype was higher in women, whereas the association between non-restorative sleep and dementia risk was stronger in men. For example, the multivariable-adjusted hazard ratio (95% CI) for sleepiness was 1.59 (1.35, 1.86) in women and 1.21 (1.04, 1.40) in men (p-interaction by sex=0.01), and the corresponding estimate for non-restorative sleep was 1.27 (1.17, 1.39) and 1.39 (1.26, 1.52) in women and men, respectively (p-interaction by sex=0.06). U-shaped associations between short/long sleep duration and dementia risk were similar in women and men. Similar sex differences were observed separately for Alzheimer’s and vascular dementia, although the associations were generally weaker for Alzheimer’s disease. In summary, most sleep health dimensions were differentially associated with dementia risk in women and men. Recognizing these sex differences may help develop sex-specific strategies to promote sleep health and moderate the risk for dementia.

